# Biomechanical induction of mild brain trauma in larval zebrafish: effects on visual startle reflex habituation

**DOI:** 10.1093/braincomms/fcad062

**Published:** 2023-03-15

**Authors:** Carolina Beppi, Marco Penner, Dominik Straumann, Stefan Yu Bögli

**Affiliations:** Neuroscience Center Zurich, University of Zurich and ETH Zurich, Zurich CH-8091, Switzerland; Department of Neurology, University Hospital Zurich and University of Zurich, Zurich CH-8091, Switzerland; Clinical Neuroscience Center, University Hospital Zurich and University of Zurich, Zurich CH-8091, Switzerland; Department of Neurology, University Hospital Zurich and University of Zurich, Zurich CH-8091, Switzerland; Neuroscience Center Zurich, University of Zurich and ETH Zurich, Zurich CH-8091, Switzerland; Department of Neurology, University Hospital Zurich and University of Zurich, Zurich CH-8091, Switzerland; Clinical Neuroscience Center, University Hospital Zurich and University of Zurich, Zurich CH-8091, Switzerland; Department of Neurology, University Hospital Zurich and University of Zurich, Zurich CH-8091, Switzerland; Clinical Neuroscience Center, University Hospital Zurich and University of Zurich, Zurich CH-8091, Switzerland

**Keywords:** concussion, linear acceleration, zebrafish, startle reflex, habituation

## Abstract

A mild traumatic brain injury is a neurological disturbance of transient or/and chronic nature after a direct blow of the head/neck or exposure of the body to impulsive biomechanical forces, indirectly affecting the brain. The neuropathological events leading to the clinical signs, symptoms and functional disturbances are still elusive due to a lack of sensitive brain-screening tools. Animal models offer the potential to study neural pathomechanisms in close detail. We recently proposed a non-invasive protocol for inducing concussion-like symptoms in larval zebrafish via exposure to rapid linearly accelerating–decelerating body motion. By mean of auditory ‘startle reflex habituation’ assessments—an established neurophysiological health index—we probed acute and chronic effects that mirror human concussion patterns. This study aimed at expanding our previous work by assessing the ensuing effects with visual—as opposed to auditory—‘startle reflex habituation’ quantifications, by using the same methodology. We observed that immediately after impact exposure, the fish showed impaired sensory reactivity and smaller decay constant, possibly mirroring acute signs of confusion or loss of consciousness in humans. By 30-min post-injury, the fish display temporary signs of visual hypersensitivity, manifested as increased visuomotor reactivity and a relatively enlarged decay constant, putatively reflecting human post-concussive sign of visual hypersensitivity. In the following 5–24 h, the exposed fish progressively develop chronic signs of CNS dysfunction, in the form of low startle responsivity. However, the preserved decay constant suggests that neuroplastic changes may occur to restore CNS functioning after undergoing the ‘concussive procedure’. The observed findings expand our previous work providing further behavioural evidence for the model. Limitations that still require addressment are discussed, advancing further behavioural and microscopic analyses that would be necessary for the validation of the model in its putative relatability with human concussion.

## Teaser

This work aimed at reproducing a mild head trauma in larval zebrafish through a non-invasive biomechanical method employing rapidly accelerating–decelerating linear movements. Through quantifications of light-induced startle reflex locomotor distance travelled and habituation, Beppi *et al.* showed transient and long-term neurophysiological changes that warrant further neuropathological investigation.

## Graphical Abstract

**Graphical Abstract:**
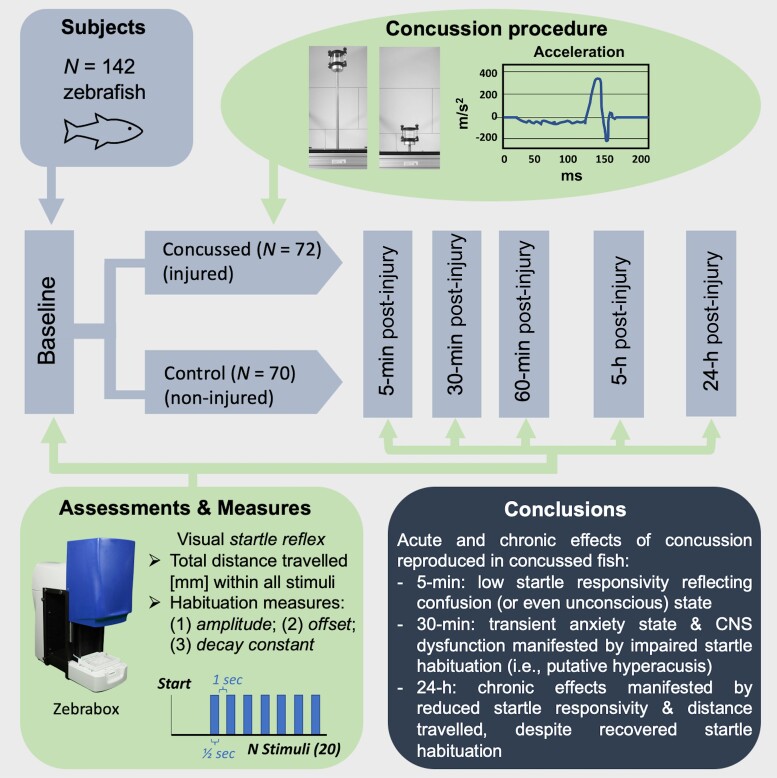

## Introduction

### Mild TBI

Concussion refers to the mildest severity subtype of traumatic brain injury (TBI)—a condition that involves compromised neurological function, after exposure to a direct head/neck impact or to impulsive body motions, which indirectly affect the brain.^1,2^ Depending on the severity of symptoms, as measured by the Glasgow Coma Scale and the duration (if present) of the post-traumatic amnesia, a TBI can be referred to as mild, moderate or severe.^3^ Another important distinguishing factor is the underlying neuropathology—namely whether there is evidence of focal injury, like in moderate-to-severe TBI cases, or rather distributed axonal/vascular microinjuries, such if the case for mild forms of TBI.^4,5^ Concussed individuals often do not display a clear underlying neuropathology that is visible on standard imaging methods, despite being symptomatic.^4,5^ For this reason, concussion remains the least understood form of TBI.

According to the Centers for Disease Control and Prevention, concussion is the most recurrent form of TBI (75%), with absolute estimations of about 0.1–0.6% of injured yearly.^6–9^ Despite being a recurring issue in contact sports, most mild TBI cases occur outside of the professional sporting context^10^ and constitute a global health concern. Due to the variable kinetic dynamics^2,4^—involving a mixture of linear and angular acceleration forces applied to the brain in different angles and extents^11^—the nature and spatial distribution of micron-size brain injuries is highly heterogenous. As such, the ensuing symptoms are also variable in the extent and nature, spanning different neurophysiological and cognitive domains, including motor, verbal, visual and auditory/balance.^1–3^ Concussed individuals typically suffer of headache, dizziness, visual or/and auditory hyperacusis, disturbed vision, compromised balance and coordination.^12,13^ The temporary disruption of ionic flow, ATP availability and metabolic function following a head impact^14^ cause transient signs and symptoms that usually resolve within a week,^15–17^ although neurological consequences may persist and turn chronic.^4^ The current understanding of mild TBI is hence limited due to the considerable inter-subject variability as well as the unavailability of sufficiently sensitive brain-screening tools.^18^

### The potential of zebrafish and the limitation of existing TBI models

Different animal species have been considered to model mild TBI and to study its underlying neuropathology, including mice, mouse and zebrafish. Zebrafish models are receiving increasing attention, due to the high degree of relatability of their functional neurobiology despite the simpler structure.^19–25^ Importantly, the relatively lower ethical burden of experimental studies with zebrafish as well as their easy maintenance and breeding^26,27^ allow the testing of reasonably large samples, hence offering higher statistical power and extent of reproducibility of the findings. In addition, microscopic screenings^28–32^ could be incorporated to define associations between structural/functional damages and the ensuing behavioural abnormalities. A large amount of research has been endeavoured to model more severe forms of TBI by means of invasive approaches such as weight drop^33^ and stab lesions^34,35^ or alternatively, mechanical and neurotoxic injuries.^36–38^

### Towards the establishment of a non-invasive zebrafish model of mild TBI

Very recently, we have proposed a less invasive model of mild TBI in larval zebrafish.^39^ By means of a custom-made motor apparatus, fish enclosed in a water-filled capsule were exposed to rapid linear decelerations that would result in concussion-like symptoms, without causing focal brain or physical damage. The ensuing effects were quantified through behavioural assessments of the auditory startle reflex habituation (SRH)—an established neurophysiological index—at different times post-impact (P-I) against a control (not impacted) group.

We observed that the impact soon after resulted in a state of excessive sensitivity to sensory stimuli with poor habituation, reflecting a temporarily deficient CNS function, while the acute symptoms resolved in a few hours, within a day since the injury chronic symptoms arose, as manifested by low motor responsivity to stimulation. By means of auditory SRH quantifications, we were thus able to probe acute and chronic effects in larval zebrafish in absence of focal injury, which resemble the symptomatic profile of human mild TBI. However, our disease model requires further behavioural and microscopic validation, to better define its relatability with its human counterpart.

### Aims and hypotheses

The aim of our study was to follow-up our first study^39^ by using the same biomechanical method to study the CNS consequences of induced rapid linear deceleration in larval zebrafish through visual—instead of auditory—SRH assessments. This would allow to relate the consequences on the visual system of larval fish to the auditory findings. We randomly assigned healthy larval zebrafish to either the impacted (*n* = 70) or control (*n* = 72) group and tested the visual SRH of both groups at baseline to define sample-specific normative response values. After the impacted group underwent the impact, both groups were re-tested at five P-I times to quantify potential intergroup differences over time. It was hypothesized that the group-specific changes relative to their baseline levels would differ significantly at all times P-I.

## Materials and methods

### Ethical approval

The experiment was conducted in line with the animal welfare guidelines of the Swiss Federal Veterinary Office. The experimental protocol was approved by the ethics committee of the Veterinary Office of the Canton of Zurich (ZH190/2020, 32971) and was also in compliance with the ARRIVE guidelines of animal research.

### Fish maintenance, breeding and egg production

Adult wild Indian karyotype *Danio rerio* zebrafish were fed and kept according to standard protocols. Following mating, the laid eggs were incubated and maintained in 1.7 L transparent breeding tanks with 28°C water under a 14- to 10-h light–dark cycle. Upon reaching 3- to 4-day post-fertilization (dpf) of age, the embryos were moved into 35 × 12 mm cell-culture plates containing E3 medium (solution in mM: 5 NaCl, 0.17 KCl, 0.33 CaCl_2_ and 0.33 MgSO_4_; Sigma-Aldrich Corp., St. Louis, MO, USA) until the following morning (first experimental test).

### Experimental apparatus

**Figure 1:**
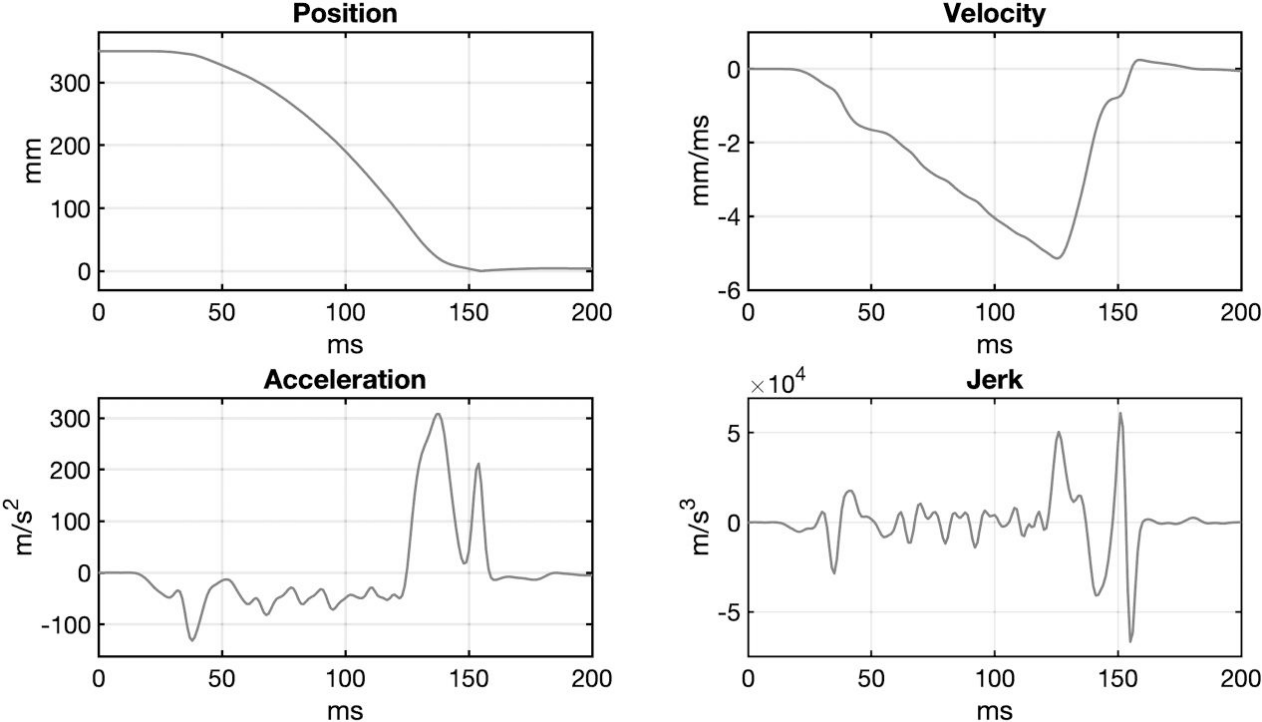
**Concussion procedure**. Features of the motion curve [position, velocity, acceleration and jerk] fed into the Linmot software and performed by the motor: duration = ∼121 ms; length = 320 mm; peak velocity = ∼5.1 m/s^2^; peak (initial) acceleration = ∼132 m/s^2^; peak (final) deceleration: 308.05 m/s^2^ (∼31.41*g), peak jerk = ∼66 785.5 m/s^2^.

The experimental apparatus was the same as that used in our previous work (Beppi *et al*.^39^). The motion curve that was fed into the LinMot software and performed by the motor is shown in Fig. 1.

### Experimental procedure

The experimental procedure was also based on our previous work (Beppi *et al*.^39^). Zebrafish larvae of 92- to 96-h past fertilization (*n* = *48*) were selected for the study. Gender could not be determined. On the testing day, they were randomly distributed to the impacted (*n* = *24*) or the control (*n* = *24*) conditions and transferred to two separate transparent air-free cylindrical capsules (each 48 × 52 mm, 60 ml) made of polystyrene and filled with E3 medium. The two groups then underwent two different treatments:

-Impacted group: The capsule containing the fish was attached to the motor to undergo linear acceleration–deceleration motion (see Beppi *et al*.^39^ for a full description of the procedure). After the injury took place, the larvae were individually inspected for any physical damage. The criteria used for indicating presence of physical injury were (i) partial loss of physical parts, (ii) presence of gross morphological changes (e.g. bending) and (iii) impeded free movement. All fish passing the visual inspection (i.e. absence of all three criteria) were moved into their 24-well plates and prepared for their 5-min P-I test. Any fish failing at least one criterion would be withdrawn from the experiment and were incubated into breeding tanks at normal/routine maintenance conditions.
-Control group: the fish were kept in the capsule, while the impacted group was undergoing the injury. This was done to expose both groups to the same change of environmental setting and control for any behavioural change related to it. In fact, exposure to a changed environmental setting is known to cause increased arousal and anxiety states (see Yokogawa *et al*.^40^ and Bai *et al*.^41^). Following impact exposure of the other group, the control fish were moved into a separate 24-well plate and tested (5-min P-I) in parallel to the impacted group.

The two groups would be again tested 30 min, 1 h, 5 h and 24 h after injury. Before each test, the health state of the impacted fish would again be assessed following the same inclusion criteria. In the time between each successive test, both groups were kept in the incubator in their respective 24-well plates, under the same conditions, until reaching the final 24-h P-I test.

The same ‘concussion-inducing’ procedure was applied three times on the same morning with a time difference of 30 min, on three different groups of 24 fish, for a total of 72 impacted fish. An approximately equal number of fish were assigned to the control group (*n* = 70). The time differences were carefully respected at each test time. The groups’ maintenance during the whole experimental procedure was blinded.

### Behavioural paradigm

The experimental paradigm consisted of a series of 20 consecutive whole-field white light stimuli of 4 kLux of 500 ms each, separated by inter-stimulus intervals (ISI) of 1000 ms. The paradigm was adapted from Beppi *et al*.^42^ During all testings, the baseline illuminance in the ZebraBox was set at environmental levels (*M* = 330 Lux), with the purpose to keep the normal light cycle of the fish unaltered, preventing arousal and changes in motility resulting from sudden illuminance changes.

### Behavioural tracking and quantitative modelling

The locomotor responses to visual stimulation were tracked using the Viewpoint ZebraBox recording system and software (ViewPoint Life Sciences, Lyon, France). The movements were quantified as distance travelled (mm) within the duration of each of the 20 stimuli (500 ms each), excluding any movements occurring in the ISIs, for each fish of both groups.

Every group’s mean total (in all stimuli) distance travelled (TDT_20_) was calculated for each of the six test times: 1 (baseline, aka 30-min pre-injury); 2 (5-min P-I); 3 (30-min P-I); 4 (1-h P-I); 5 (5-h P-I); and 6 (24-h P-I). The individual fish TDT of the five tests performed after the impact was then normalized subtracting the respective baseline TDT, to obtain a measure of (baseline-relative) change in TDT. This measure was then statistically tested for differences between groups across time. Only fish that survived the impact—without showing evidence of physical/motor injury—and performed all six testings were included in the analyses.

Data bootstrapping (*n* = 500, in-built MATLAB function: bootstrp) was applied on the habituation curve (distance travelled over stimuli) of each individual fish (of both groups) to obtain a population-level distribution of behaviour. Based on the SRH model proposed by Beppi *et al*.,^42,43^ a first-order exponential (in-built MATLAB function: lsqcurvefit) was fitted into the bootstrapped data to extract ‘amplitude’, ‘decay constant’ and ‘offset’—the three descriptive measures of startle habituation (for a definition, please refer to Beppi *et al*.^39,42,43^). These measures were obtained with the same procedure for the baseline, 5-min, 30-min and 24-h tests. The decay constant values were winsorized: all negative (and hence impossible) values were corrected as being equal to 0 (minimum), and all values >10 were corrected as being equal to 10 (i.e. the maximum rounded *D*_c_ value of the model).

### Theoretical definition of mild TBI in zebrafish

For a definition of how the movement kinematics of deceleration in our experiments were determined to induce a mild TBI (as opposed to moderate or severe), please refer to our previous work (Beppi *et al*.,^39^).

### Statistical analyses

MATLAB R2021a (The MathWorks Inc., Natick, Massachusetts, USA) and SPSS Statistics version 27.0 (IBM Corp., Armonk, New York, USA) were used for conducting statistical analyses. A mixed two-way ANOVA was run to test differences between the two groups in (baseline-relative) change in TDT, across the different re-test times. The first independent variable consisted of the group (impacted or control), while the second repeated measures IV was time, with five levels: 5-min, 30-min, 60-min, 5-h and 24-h P-I. The dependent variable consisted of the TDT (continuous measure).

## Results

### Initial inspection after the impact

Freely swimming zebrafish larvae enclosed in a polystyrene capsule filled with E3 medium were exposed to a rapid linearly accelerating–decelerating motion with a peak deceleration of ∼308 m/s^2^ (∼31.4 g). After this single ‘concussive’ event, all fish were alive and were considered physically healthy (i.e. not displaying a focal brain or body injury) upon visual inspection. No fish was thus excluded from the analysis.

### Startle response habituation before and after impact

**Figure 2:**
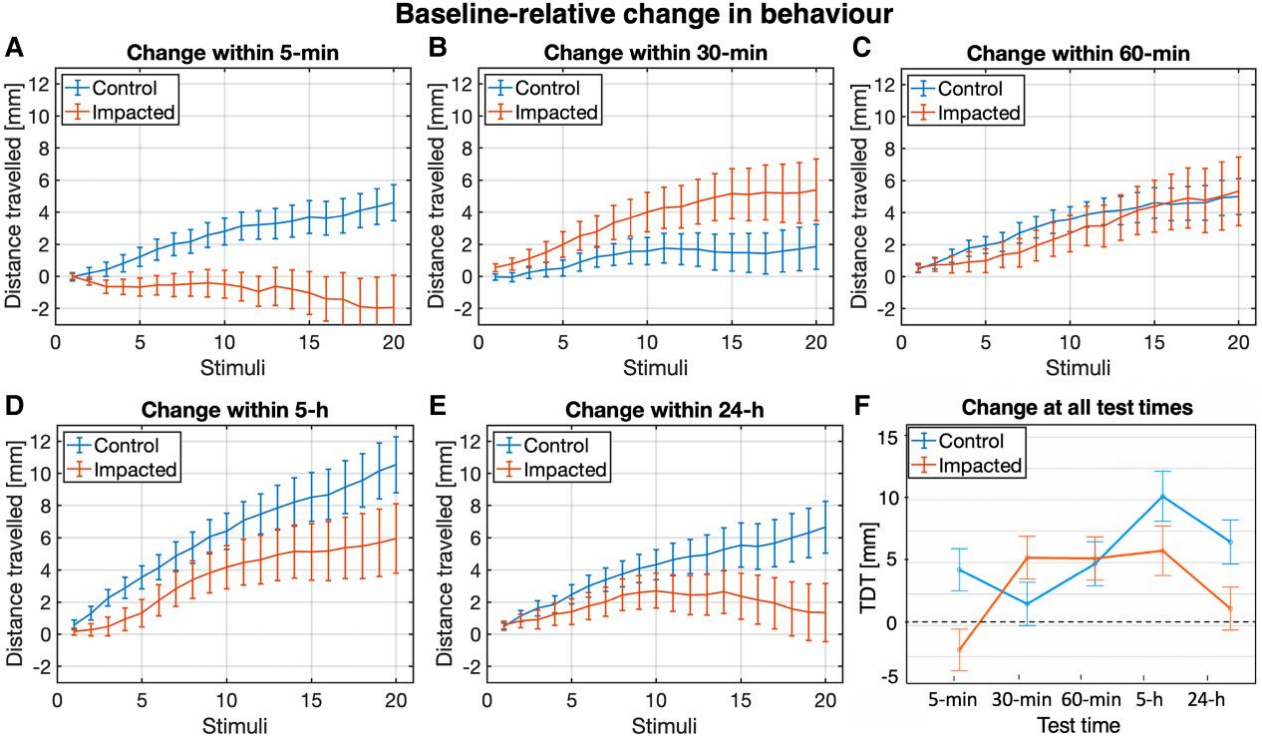
**Change in startle responses over test times.** In **A**, **B**, **C**, **D** and **E**, change in distance travelled (±1 SE) of each group relative to their respective baseline levels, at each stimulus, for each re-test time. In **F**, change in TDT (±1 SE) of each group relative to their respective baseline levels, at each re-test time (2: 5 min, 3: 30 min, 4: 60 min, 5: 5 h, and 6: 24 h). A 2 × 5 mixed-design ANOVA was performed to test if the baseline-relative change in TDT at all re-test times (5 min, 30 min, 60 min, 5 h and 24 h) would differ between the control group (*n* = 70) and the impacted (*n* = 72). As the data did not fulfil the parametric test assumptions of sphericity and homoscedasticity, corrected *F* statistics (Greenhouse–Geisser, Welch’s or Brown–Forsythe) were considered to determine statistical significance. No significant main effect of group was found (*P* > 0.05). The main effect of time was instead significant [*F*(3.707,518.937) = 7.114, *P* < 0.001]. However, the significant interaction between group and test time [*F*(3.707,518.937) = 5.158., *P* < 0.001] indicated that the effect of time was different for the control and impacted group. Two one-way repeated-measures ANOVAs were run to break down the interaction effect, analysing the effect of time (five levels) for the control and impacted groups separately. A significant effect of time [*F*(4,276) = 8.171, *P* < 0.001] was found on the baseline-relative change in TDT of the control group. Pairwise comparisons further indicated that the increase in TDT relative to baseline was significantly larger at Test Time 5 (5-h P-I, *M* = 10.28, SE = 1.78) than at Test Time 2 (5-min P-I, *M* = 4.44, SE = 1.15, *P* = 0.005), Test Time 3 (30-min P-I, *M* = 1.71, SE = 1.43, *P* < 0.001) and Test Time 4 (60-min P-I, *M* = 4.91, SE = 1.14, *P* = 0.037). The baseline-relative increase in TDT at Test Time 6 (24-h P-I, *M* = 6.64, SE = 1.65) was also significantly larger than at Test Time 3 (*P* = 0.03). The results of the ANOVA for the impact group also revealed a significant effect of time [*F*(3.296,234.048) = 5.134, *P* = 0.001] over the baseline-relative change in TDT. Pairwise comparisons further indicated a significantly larger (baseline-relative) increase in TDT at Test Time 4 (*M* = 5.34, SE = 2.14, *P* = 0.033) and Test Time 5 (*M* = 5.95, SE = 2.16, *P* = 0.032), compared to Test Time 2 (*M* = −1.95, SE = 2.03), when the TDT instead decreased relative to baseline. No further statistical differences at pairwise levels were found.

The SRH tested before (baseline) the impact was subtracted from the SRH tested at different times after the injury. The trajectory of change in cumulative distance travelled over 20 visual stimuli for the impacted larvae (*n* = 72) was different from that of the control larvae (*n* = 70), as shown in Fig. 2. Five-minute P-I, the TDT_20_ of the control group was markedly larger compared, while that of the impacted group was lower, compared to their respective baseline (Fig. 2A and F). Half-an-hour P-I, the impact group’s (baseline-relative) change TDT rises significantly, beyond the control group’s level (Fig. 2B and F). One hour after the impact, the groups show similar TDT levels (Fig. 2C and F), while 1 day later, the TDT_20_ of the impacted group was significantly smaller (Fig. 2E and F). Details about the statistical tests performed and precise statistical numbers are reported in the legend of Fig. 2. The distance travelled during each stimulus at acute P-I times for both groups are shown in Supplementary Fig. 1.

### Exponential fitting of cumulative distances travelled

**Figure 3:**
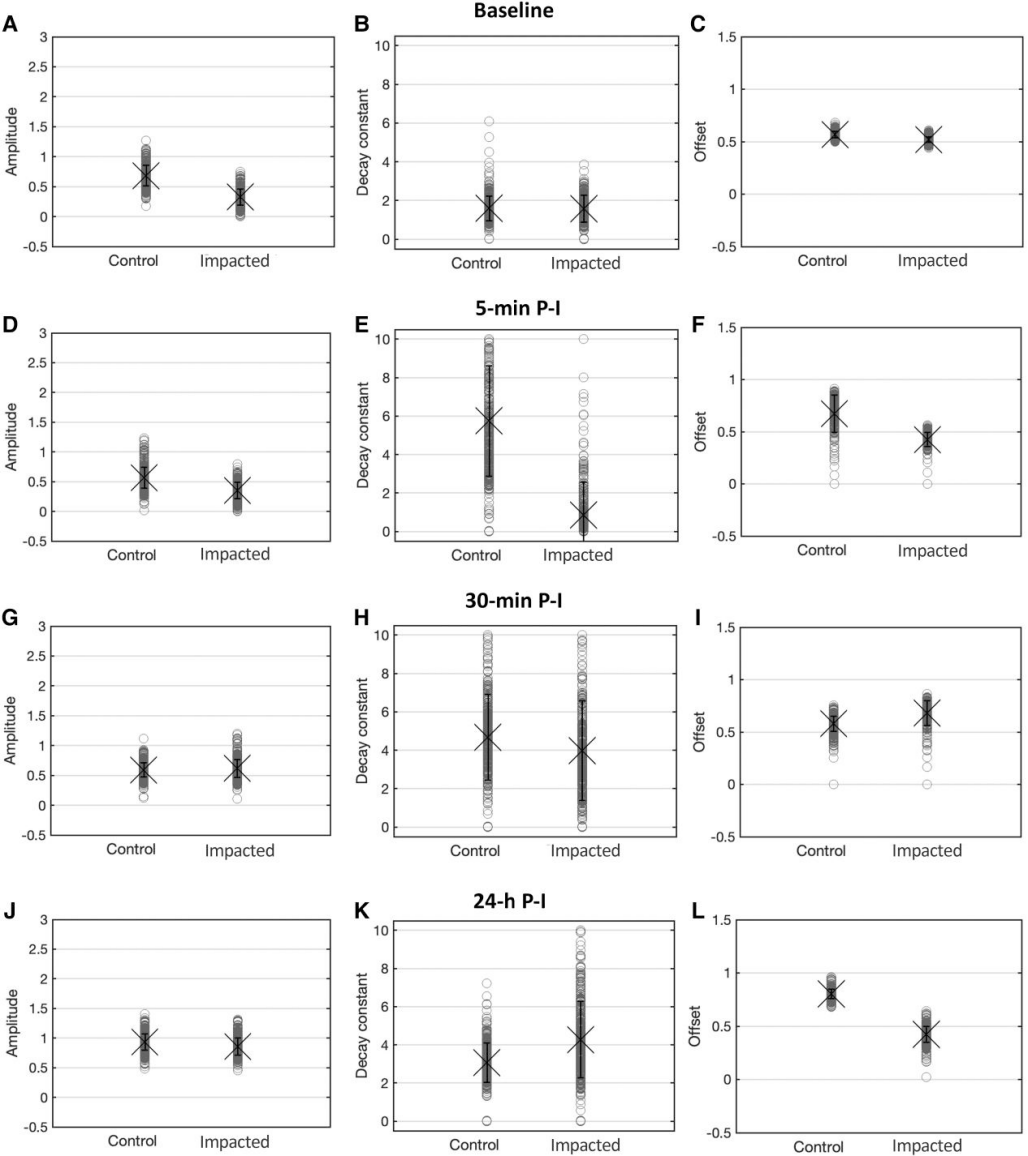
**Population-level habituation descriptives.** Descriptive statistics of the mean (±1 SE) amplitude (**A**, **D**, **G**, **J**), decay constant (**B**, **E**, **H**, **K**) and offset (**C**, **F**, **I**, **L**) of 500 bootstraps of locomotor distance travelled over the 20 visual stimuli—fitted to a single exponential—for the control and impacted groups, at baseline, 5-min, 30-min, 60-min and 24-h P-I.

The habituation curves (locomotor distance travelled over the 20 visual stimuli) of 500 bootstraps were fitted to a single exponential to determine which SRH measure (amplitude, decay constant and offset) underlined the observed TDT_20_ differences between the groups (Fig. 3). Five-minute P-I, the high TDT_20_ of control fish was accompanied by an enlarged decay constant and offset (steady-state responsivity), against the slight *D*_c_ reduction of the impacted fish (Fig. 3E and F). Increased decay constant and offset underlie the TDT recovery of the impacted group fish to control levels within 30-min P-I (Fig. 3H and I). One day (24 h) after the injury, the decay constant of the two groups does not show gross differences (Fig. 3K). The smaller offset (Fig. 3L) of the impacted group underlies its lower reactivity to visual stimulation.

## Discussion

Mild TBI is the most recurrent form of head trauma within and outside the professional sporting context and constitutes a global health concern. The current unavailability of sensitive brain-screening tools has limited the understanding of the pathomechanisms following mild TBI. Existing zebrafish models offered the opportunity to experimentally reproduce moderate-to-severe head impacts to study the consequent neurobiological pathomechanisms. However, milder TBI forms (not using invasive methods) have not been modelled insofar.

Recently, we have proposed^39^ a non-invasive protocol for inducing linearly accelerating–decelerating motion forces in larval zebrafish that result in concussion-like symptoms, in absence of gross physical or focal brain damage. By assessing the auditory SRH, we were able to probe CNS dysfunctions in larval zebrafish that mimic the acute and chronic effects observed in human mild TBI. The model however requires further validation that can better define the behavioural effects using different paradigms, as we will later discuss. Also, the biochemical underlyings of the observed deficits, as well as microscopic evidence excluding physical microtraumas, are yet to be conducted to support a univocal relatability of the model with human concussion.

In this study, we used the same biomechanical procedure^39^ to quantify the resulting neurological deficits of induced rapid linear deceleration by mean of visual (instead of auditory) SRH assessments. We hence aimed at assessing how the visual system responds to a similar neurological trauma, relatively to the auditory system.^39^ Healthy larval zebrafish were randomly assigned to either the impact (*n* = 72) or control (*n* = 70) condition and tested on a visual SRH task before injury and again at five times P-I, to track differences between groups across time. We predicted that the baseline-relative change in the SRH profile and TDT would differ significantly between groups at all test times.

Overall, the results confirm our hypotheses, revealing that the effect of time was different for the control and impacted fish. Comparably to our previous work on auditory SRH,^39^ the control fish displayed an overall pattern of baseline-relative increase in TDT over time, evidencing a normal path of developmental growth. The baseline-relative change in TDT of the impacted fish showed relatively abnormal patterns.

A number of comparative observations between the groups can be made, for each single test time. When tested 5-min P-I, the control group shows a marked growth in TDT and decay constant. This suggests that the mere change in environmental setting (capsule)—without impact exposure—has acted as environmental stressor (see e.g. Bai *et al*.^41^) inducing a high arousal and anxiety state, which resulted into increased startle responses and impaired habituation. Interestingly, the impacted group displays a reduced baseline-relative TDT and smaller decay constant (slower decay). The findings suggest that the impact caused a transient CNS dysfunction evidenced by a low level of startle responsivity, which might mirror the acute signs of confusion—and possibly, loss of consciousness—occurring in humans immediately after undergoing a concussion.

One might further consider that the concussion procedure itself might also have induced some forms of anxiogenic/arousal behaviours. In fact, the phenomenon of an ensuing hypoactivity followed by a relative hyperactivity—albeit with a slightly different time scale—was described by Yokogawa *et al*.^40^ Such effects could be better quantified by having an additional comparison condition where the fish are exposed to a lower impact—under the threshold for causing CNS-level effects—and compared to controls. Any differences in behaviour would be attributable to the concussion procedure.

At Time 3 (30-min P-I), the impacted fish show a higher (baseline-relative) increase in TDT compared to the respective baseline-relative increase of the control group. Their decay constant increased up to control levels. The behaviour seems to mirror visual hypersensitivity—a commonly reported symptom in human concussion.^11^ The motor responses reach control levels within 60-min P-I.

By the fifth hour P-I, the (baseline-relative) change in TDT of the impacted group drops remarkably and worsens within 24-h P-I, evidencing a chronic CNS dysfunction. The effects arguably mirror human post-concussive syndrome (PCS).^44^ While in fact mild TBI signs and symptoms are mostly transient and remitting in nature, a concussion can result in persistent neurological disturbances.^45^ Despite the reduced motor reactivity 1 day after the injury, the decay constant of habituation—despite a slightly lower offset—seems to be spared, resembling control levels. This might reflect brain plasticity processes apt to restore gross CNS functions following cellular-, molecular- or metabolic-level disruptions.^19–21,46^ In fact, abnormal brain activity has been detected in PCS cases,^47,48^ while recovering individuals display compensatory activity patterns during demanding cognitive tasks^45,49^ that reflect plastic neural reorganizations. Future studies might consider performing calcium imaging and microscopy analyses to probe cellular and molecular changes (i.e. in serotonergic activity), to test this hypothesis. The optical translucency of zebrafish at larval stages^26,30,31^ in fact allows detailed non-invasive brain screenings.

Contrary to our expectations, the results largely mirror the observations made with the vibratory SRH paradigm.^39^ Similar gross effects on SRH—as assessed by the changes in decay constant over time—independently from the modality of the sensory stimulus. This suggests that habituation is a fundamental neurophysiological (CNS) function index, for which the sensory input is largely irrelevant. Habituation in fact classifies more as memory and non-associative learning, than motor function. To further separate the memory and learning functions from the motor function, one might think of tracking free locomotion continuously over the time frame of 24–48 h (e.g. using the approach of Basnet *et al*.^50^). Then, one would divide the data into time bins of a few hours and compare the groups for the TDT within each time bin. The absence of between-group differences in this free locomotion test would exclude any motor effects.

However, some differences between the effects observed for the light- and vibration-induced SRH are to be acknowledged. In our previous study,^39^ no deficit was observed at Time 2 (5 min), when instead signs of sensory hypersensitivity were present. In contrast, in this study, signs of visual hypersensitivity appeared 30-min P-I. Moreover, we previously reported chronic signs within 70-min P-I, while in this study, they arose between 60-min and 5-h P-I. The differences may be accounted for by the different sensory modalities of the SRH paradigms used, meaning that the visual and auditory systems may be affected differently by mild TBI.

A second reason might reside in the different kinetics of the impact incurred by the impacted fish in our two studies. Relative to our previous work,^39^ the initial acceleration was higher, and the peak deceleration was followed by a second weaker peak, which could not be completely prevented by the motor, which was due to inertial force. This might have constituted a second concussive event, resembling a coup–contrecoup kind of impact. Whether this caused the ‘refraction period’ at 5-min P-I, during which transient confusion and dysfunctions were observed, remains an open question. This further ‘phase’ may also be responsible for the delayed onset (within 30 min) of sensory hypersensitivity, which in our previous work^39^ was instead observed within 5-min P-I. We hence hypothesize that the jerk (i.e. the derivative of acceleration) may be a more relevant factor contributing to the pathological events following impact exposure.

For future research purposes, one could design a set of movement profiles with systematic variation of given parameters (e.g. step-wise increments of peak acceleration, deceleration or jerk) to comparatively assess their effect on the SRH of larval zebrafish. Such profiles could be used to assess the consequences at CNS level and define a ‘tolerance curve’ describing the amount of ‘cumulative stress’ (as linear force) the brain can tolerate, before undergoing irreversible damage. Such design would also allow to control (balance out) the effect of induced anxiety across groups exposed to different motion curves.

## Conclusions

We conclude that SRH decay constant is mostly sensitive to acute effects occurring within the first hour after impact exposure, when the fish transfer from initial state of confusion (low responsivity and slow decay constant 5-min P-I) to hypersensitivity (high responsivity and fast decay constant within 30 min). Chronic deficits/symptoms are instead better represented by mere SRH TDT quantifications. The SRH can hence be considered a sensitive marker for transient as well as chronic changes in neurological states, in line with our previous work with larval zebrafish^39^ as well as recent human startle findings in humans.^51^

This study—added to our previous work^39^—constitute a step forward towards the establishment of a behavioural model of mild TBI in larval zebrafish through the application of rapid linear forces on zebrafish larvae enclosed in water-filled capsules. This model can be regarded as being parsimonious in its ability to non-invasively reproduce concussion-like transient and chronic symptoms in larval zebrafish, while maintaining simplicity. As such, it contributes to the understanding of a pathology, while minimizing the ethical burden of animal experimentation for translational purposes.

Further model validation is however required: the transient and chronic neurophysiological changes—as assessed by the visual SRH—warrant additional investigation not only to probe eventual diffuse micrometre- to nanometre-level neuropathological changes (i.e. vascular or axonal) that might underlie the observed effects but also to exclude non-neural pathologies and to study putative plasticity mechanisms of recovery.

As repeated concussions are common in professional sporting contexts and are cause of more severe negative outcomes,^52,53^ lines of further research might consider investigating the outcome of sub-concussive impacts in larval zebrafish, as done with the mouse (e.g. Lavender *et al.*^54^ and Pham *et al*.^55^), by executing the same linear movement profile twice, at varying time intervals. Such experiments would be valuable to define the critical time window in which the brain is at the highest vulnerability to irreversible damage.

One might furthermore consider studying the effect of rapid angular—as opposed to linear—accelerations/decelerations varying in trajectory parameters, to model more severe and distributed injuries.^56,57^ Finally, additional well-established sensory-processing testings in larval zebrafish, including the optokinetic reflex (e.g. Rinner *et al*.^58^ and Scheetz *et al*.^59^) and the vestibulo-ocular reflex (e.g. Mo *et al*.^60^ and Sun *et al*.^61^), could be assessed—in parallel with the SRH—to provide insights into how the oculomotor and vestibular systems are affected relationship between sensory systems how relate to SRH.

## Supplementary Material

fcad062_Supplementary_Data

## Data Availability

The raw data that support the findings of this study are available from the corresponding author upon reasonable request.

## Abbreviations

ISI =: inter-stimulus interval
P-I =: post-impact
PCS =: post-concussive syndrome
SRH =: startle reflex habituation
TBI =: traumatic brain injury
TDT =: total distance travelled
