# Supplementary material for: Biomechanical induction of mild brain trauma in larval zebrafish: effects on visual startle reflex habituation
Source: Brain Commun. 2023 Mar 15;5(2):fcad062. doi: 10.1093/braincomms/fcad062 (PMC10065185; doi:10.1093/braincomms/fcad062)
Supplement: fcad062_Supplementary_Data [file fcad062_supplementary_data.pdf]

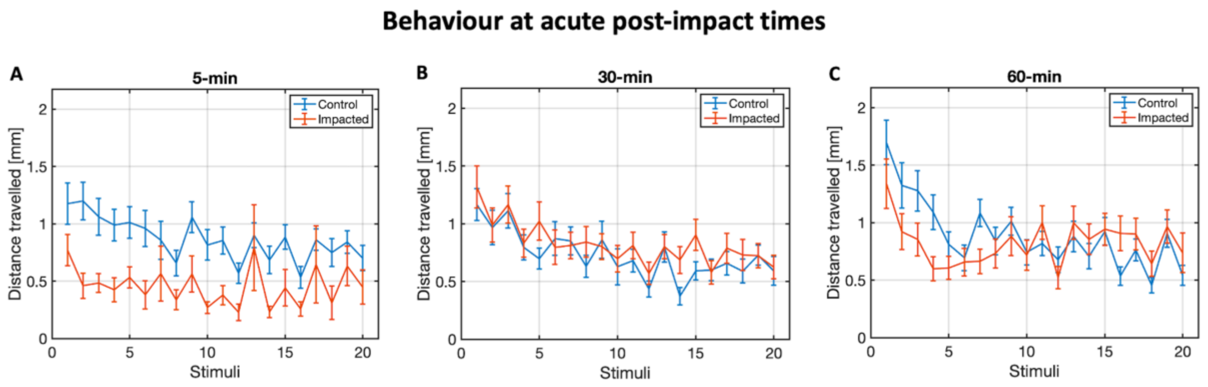

Supplementary Figure 1 | Startle responses at acute test-times. Distance travelled (+/- 1 SE) of each group, at each stimulus, at the following re-test times: 5-min (A), 30-min (B), 60-min (C).
